# Rebound of residual plasma viremia after initial decrease following addition of intravenous immunoglobulin to effective antiretroviral treatment of HIV

**DOI:** 10.1186/1742-6405-8-21

**Published:** 2011-06-28

**Authors:** Tomas Mellberg, Veronica D Gonzalez, Annica Lindkvist, Arvid Edén, Anders Sönnerborg, Johan K Sandberg, Bo Svennerholm, Magnus Gisslén

**Affiliations:** 1 Department of Infectious Diseases, University of Gothenburg, Sahlgrenska University Hospital, Sweden; 2 Center of Infection Medicine, Department of Medicine, Karolinska Institute, Stockholm, Sweden; 3 Department of Laboratory Medicine, Division of Clinical Microbiology, Karolinska Institute, Stockholm, Sweden; 4 Department of Infectious Diseases, Karolinska Institute, Stockholm, Sweden; 5 Department of Clinical Virology, University of Gothenburg, Sahlgrenska University Hospital, Sweden

## Abstract

**Background:**

High dosage of intravenous immunoglobulin (IVIG) has been observed as a possible activator of HIV gene expression in latently infected resting CD4^+ ^T-cells, leading to a substantial decrease in both the reservoir and the residual plasma viremia when added to effective ART. IVIG treatment has also been reported to expand T regulatory cells (Tregs). The aim of this study was to evaluate possible long-term effect of IVIG treatment on residual viremia and T-lymphocyte activation.

**Methods:**

Nine HIV-infected subjects on effective ART included in a previously reported study on IVIG treatment were evaluated 48-104 weeks after therapy. In addition, 14 HIV-infected controls on suppressive ART were included. HIV-1 RNA was analyzed in cell-free plasma by using an ultrasensitive PCR-method with a detection limit of 2 copies/mL. T-lymphocyte activation markers and serum interleukins were measured.

**Results:**

Plasma residual viremia rebounded to pre-treatment levels, 48-104 weeks after the initial decrease that was observed following treatment with high-dosage IVIG. No long-term effect was observed regarding T-lymphocyte activation markers, T-regulatory cells or serum interleukins. In a post-hoc analysis, a correlation between plasma HIV-1-RNA and CD4^+ ^T-cell count was found in both IVIG-treated patients and controls.

**Conclusions:**

These results indicate that the decrease in the latent HIV-1 pool observed during IVIG treatment is transient. Although not our primary objective, we found a correlation between HIV-1 RNA and CD4^+ ^T-cell count suggesting the possibility that patients with a higher CD4^+ ^T-cell count might harbor a larger residual pool of latently infected CD4^+ ^T-cells.

## Background

The latency of HIV-1 in resting CD4^+ ^T-lymphocytes constitutes a major obstacle for the eradication of virus in patients on otherwise effective antiretroviral therapy (ART)[1]

High dosage of intravenous immunoglobulin (IVIG) has been proposed as a possible activator of HIV gene expression in latently infected resting CD4^+ ^T-cells. In a previous study, patients on ART were given 30 g immunoglobulin intravenously per day for 5 consequent days (0,4 g/kg). When IVIG was added to effective ART, a substantial decrease in residual plasma viremia and in the virus reservoir was observed in a majority of subjects[2]. The latent HIV-1 pool in resting CD4^+ ^T-cells decreased with in median 68% after addition of IVIG. The reservoir decreased in five, whereas no decrease was found in two subjects with detectable virus. Plasma HIV-1 RNA ≥ 2 copies/mL was detected in five of seven subjects at baseline, but in only one at follow-up after 8-12 weeks. The decrease in the latent HIV-1 pool and the residual plasma viremia was preceded by an initial transitory low-level increase in plasma HIV-1 RNA during IVIG treatment that likely originated from a release of virus from the latent reservoir[2,3]. Viral clones from plasma clustered together with virus from latently infected memory T-cells as measured by single genome sequencing (SGS) of the *gag *region. Furthermore, the magnitude of the increase in HIV-1 RNA in plasma correlated with the size of the latent HIV-1 pool. IVIG also resulted in a consistent increase of CD25^+^CD127^- ^regulatory T-cells (Tregs) from median 1.4 (IQR: 0.96-2.2)% to 2.3 (1.3-3.3)%, in all subjects after IVIG treatment, *p *= 0.0036[2]. The aim of this follow-up study was to investigate if the observed decrease of residual viremia and increase of Tregs was transient or maintained over time. In addition we aimed to analyze the relationship between low-level residual viremia and immune activation markers during effective ART.

## Materials and methods

### Patients

The nine patients that were included in the previously presented IVIG-study[2] were sampled again, 48-104 weeks after treatment with IVIG. They had all been on suppressive ART ≥2 years and had plasma HIV-1 RNA levels < 50 copies/mL ≥1,5 years before the IVIG therapy was given in accordance with the study protocol. HIV-1 RNA remained < 50 copies/mL in all subjects between the time of the previous study and new follow-up sampling. In addition, 14 HIV-infected subjects on suppressive ART and fulfilling the same inclusion criteria as stated above were included as controls. The control group was not part of the original study. Patient characteristics are shown in Table 1. All subjects provided written informed consent, and the study was approved by the Research Ethics Committee at the University of Gothenburg.

**Table 1:** Patient characteristics

Patient Number* (Age, sex)	ART regimen	Duration (months)			CD4^+ ^T-cell count (cells/μL)	
				
		Total time under treatment	Time with HIV-1 RNA < 50 copies/mL	Time in treatment after IVIG administration	Current	Nadir
1 (65,M)	ABC+3TC+EFV	103	96.1	24.3	470	180
2 (53,M)	TDF+FTC+EFV	147.6	105.3	21.6	790	200
3 (35,M)	ABC+ZDV+3TC+LPV/r	76.6	73.6	20.5	180	20
4 (36,F)	ABC+3TC+ATV/r	76	71	21.5	430	50
5 (58,M)	3TC+ABC+LPV/r	145.6	84.3	11.7	150	40
6 (36,M)	ZDV+3TC+EFV	132.7	48.3	12.4	240	120
7 (43,M)	TDF+FTC+LPV/r	99	32	10.6	550	40
8 (45,M)	3TC+ABC+NVP	95.4	87.2	12.1	310	30
9 (43,M)	TDF+FTC+ATV/r	92.6	79.3	12.3	640	90
Median		99	79.3	12.4	430**	50
C1 (38,F)	3TC+ABC+ATV/r	56.7	55.9		910	176
C2 (45,M)	ZDV+3TC+SQV/r	88.4	75.1		730	230
C3 (26,F)	3TC+ABC+EFV	47	46.2		470	240
C4 (47,F)	TDF+FTC+LPV/r	84.6	80		1000	20
C5 (44,F)	ETV+ATV/r+RAL	94.7	80.4		530	42
C6 (42,M)	ZDV+FTC+EFV	55.8	50.3		430	80
C7 (39,F)	3TC+ABC+ATV/r	104	110.2		480	17
C8 (61,M)	TDF+FTC+EFV	134	78.5		530	340
C9 (42,F)	3TC+ABC+LPV/r	132	128.6		510	40
C10 (56,M)	ZDV+3TC+ABC	153.4	140.4		750	45
C11 (36,F)	3TC+ABC+LPV/r	100.4	93.1		700	200
C12 (43,M)	3TC+ABC+LPV/r	32.1	24.3		550	190
C13 (46,F)	TDF+FTC+EFV	83.6	71.9		330	40
C14 (37,F)	ZDV+3TC+LPV/r	62.3	58.3		720	140
Median		86.5	76.8		540**	110

* Controls are denoted C1-C14

** CD4^+ ^T-cell count differed between groups (p = 0.05)

ABC, abakavir; ATV/r, atazanavir/ritonavir; EFV, efavirenz; ETV, entecavir; FTC, emtricitabine; LPV/r, lopinavir/ritonavir; NVP, nevirapine; RAL, raltegravir; SQV/r, saquinavir/ritonavir; TDF, tenofovir; ZDV, zidovudine; 3TC, lamivudine;

## Methods

### Quantification of HIV-1 RNA in plasma

HIV-1 was quantified in cell-free plasma by a previously described modified version of the Roche Amplicor Monitor Test (Version 1.5, Roche Diagnostic Systems, Hoffman-La Roche, Basel, Switzerland) with a lower dynamic detection limit of 2 copies/mL[4]. The method allows detection by signal below 2 copies but cannot differentiate an exact number of copies below this limit. In graphs, samples with positive HIV-1 RNA PCR signal below 2 copies/mL were denoted as 1,5 copies/mL whereas samples with negative HIV-1 RNA PCR signal were denoted as 1 copy/mL. All plasma HIV-1 RNA samples with values below the quantitative detection limit but with a PCR signal were counted as < 2 copies/mL in calculations.

### Cytokine analysis

Plasma samples from all patients were analyzed for the presence of IL-2 and IL-7 cytokines on a Luminex 100™ System (Luminex Corp, Austin, TX, USA). The procedure is described in a protocol supplied with the IL-2 and IL-7 Human Singleplex Bead Kits (Invitrogen). Abs from the two kits were combined, and undiluted plasma samples were thoroughly mixed, centrifuged, and filtered prior to analysis.

### T-cell assays, flow cytometry, and mAbs

Peripheral blood CD4^+ ^and CD8^+ ^T-cell counts were measured by direct immunofluorescence in a flow cytometer. Flow cytometry was used for phenotypic analysis of lymphocytes and measurement of T-lymphocyte activation markers CD38 and HLA-DR. For each sample, 7 × 10^5 ^freshly isolated PBMC were stained for surface markers in a 96-well v-bottomed plate on ice for 30 min, washed three times in PBS with 1% FCS, before permeabilization with Foxp3 staining buffer (eBiosciences) at 4°C for 30 min. Cells were then washed with Permeabilization buffer and stained for intranuclear Foxp3 for 30 min, washed three times, and resuspended in Permeabilization buffer. For intracellular staining of cytokines, cells were stained for surface markers before permeabilization with Perm/Fix solution (BD Biosciences) at 4°C for 20 min. Cells were then washed with Perm/Wash solution and stained for intracellular IFNγ, MIP-1β, IL-2, and TNFα for 30 min, washed three times, and resuspended in CellFix solution. Multicolor flow cytometry data was acquired on a CyAn ADP instrument (Dako)[5]. Data were analyzed using FlowJo software (Tree Star, Ashland, OR, USA). The HIV-Gag p55 peptide pool (JPT Peptide Technologies, Berlin, Germany) was used to study the HIV-1-specific responses, and a CMV, EBV, and Flu (CEF) control peptide pool, as well as Staphylococcal Enterotoxin B (SEB) (SIGMA-Aldrich Logistic GmbH, Schnelldorf, Germany), were added as positive controls. The PBMCs were plated at a concentration of 1 × 10^6 ^cells/well, along with peptides at a final concentration of 2 μg/mL per peptide in the pool, and incubated at 37°C for 12 hrs. As a negative control, cells were incubated with medium only to determine the background responses for each patient. The following mAbs were used: anti-CD3 PE-Cy7, anti-CD8 PerCP, anti-CD25 PE, anti-CD38 FITC PE-Cy7, anti-CD127 Alexa647, anti-HLA-DR APC-Cy7, anti-IFNγ FITC, anti-MIP-1β PE, and anti-IL-2 APC, all from BD Biosciences (San Diego, CA, USA). TNFα Pacific Blue from eBioscience, Anti-CD3 Pacific Blue from Dako (Copenhagen, Denmark), anti-Foxp3 A488 from Biolegend, anti-CD4 Qdot 605 and Aqua Live/Dead cell exclusion marker from Invitrogen.

### Statistics

Statistical analyses were performed by using Graphpad Prism 5.0 for Mac OS X. The Mann-Whitney U-test was used for comparisons between two independent groups, Wilcoxon Signed Rank test for pair wise comparisons, and Spearman's Rank Correlation Coefficient for evaluations of correlations. The One-way ANOVA-test was used for calculations of repeated measures.

## Results

### Plasma HIV-1 RNA

**Figure 1:**
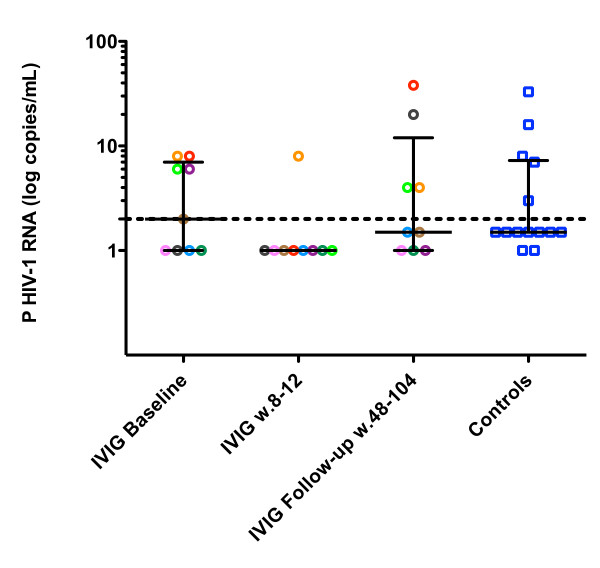
**The effect of intravenous immunoglobulin (IVIG) on plasma HIV-1 RNA**. Rebound of plasma HIV-1 RNA to pre-treatment levels at follow-up after initial decrease 8-12 weeks after treatment with IVIG. At follow-up six out of nine patients had detectable levels of plasma HIV-1 RNA (median < 2 copies/mL, IQR < 2-12 copies/mL, mean 8 copies/mL ± SD 12.8) compared to one patient 8-12 weeks after IVIG treatment (median < 2 copies/mL, IQR < 2 copies/mL, mean 1.7 copies/mL SD ± 2.3). No difference in plasma HIV-1 RNA between treated (median < 2 copies/mL IQR < 2-12 copies/mL, mean 8 copies/mL SD ± 12.8) and controls (median < 2 copies/mL, IQR < 2-7.25 copies/mL, mean 5.7 copies/mL SD ± 8.9) at follow-up. Quantitative detection limit (2 copies/mL) is marked by grid line.

Plasma residual viremia rebounded to pre-treatment levels at patient follow-up, 48-104 weeks after the initial decrease observed at 8-12 weeks after treatment with high-dosage IVIG. At follow-up, six out of nine patients (subjects 1-3 and 7-9) had detectable levels of plasma HIV-RNA compared to one patient (subject 7), 8-12 weeks after IVIG treatment. Three of the subjects (subjects 4-6) had undetectable levels of plasma HIV-1 RNA at follow-up and another two (subjects 2 and 3) had detectable plasma HIV-1 RNA below 2 copies/mL, Figure 1. There was no significant difference in plasma HIV-1 RNA between IVIG-treated subjects (median < 2 copies/mL, IQR < 2-12 copies/mL) and controls (median < 2 copies/mL, IQR < 2-7.2 copies/mL) at follow-up, Figure 1.

### T-cell analyses

We did not detect any significant difference in Treg (CD4^+^CD25^+^CD127^-^FoxP3^+^) proportion of peripheral blood CD4^+ ^T-cells between IVIG-treated patients (median 0.86%, IQR 0.60-1.21%) and controls (median 0.91%, IQR 0.79-1.33%).

Different antibodies were used in the previous study to identify Tregs making direct comparisons of results from the period during IVIG treatment and follow-up impossible.

No change in peripheral CD4^+ ^T-cell count was detected from baseline to follow-up or during the IVIG-treatment. A lower CD4 count for IVIG-treated patients (median 430 cells/μL, IQR 210-595) compared to controls (median 540 cells/μL, IQR 478-755) was observed at follow-up (p = 0.05). The CD4^+ ^T-cell nadir was also lower in patients treated with IVIG (median 50 cells/μL, IQR 35-150) than in controls (median of 110 cells/μL, IQR 40-208), however, the difference was not statistically significant. CD4^+ ^T-lymphocyte activation as measured by expression of CD38 was lower for IVIG-treated patients at follow-up compared to controls (44.5% IQR 37.7-58.6 for IVIG treated, 58.5% IQR 47.9-70.2 for controls, p = 0.04). CD38 expression of CD8^+ ^T-lymphocytes was also lower for IVIG-treated patients (10.1% IQR 7.2-17.2% for IVIG treated, 15.2% IQR 12.5-23.8% for controls, p = 0.07). However, CD38-expression on CD4^+^and CD8^+ ^T-cells was stable during IVIG-treatment with no difference compared to baseline levels within the IVIG-treated group. Therefore, results are probably a consequence of differences between groups irrespective of IVIG treatment. HLA-DR expression of CD4^+ ^or CD8^+ ^T-lymphocytes did not differ between the groups. No effect was observed on HIV-specific CD8^+ ^T-cell responses against Gag peptide pools when IVIG treated patients were compared with controls.

### Correlations with plasma HIV-1 RNA

**Figure 2:**
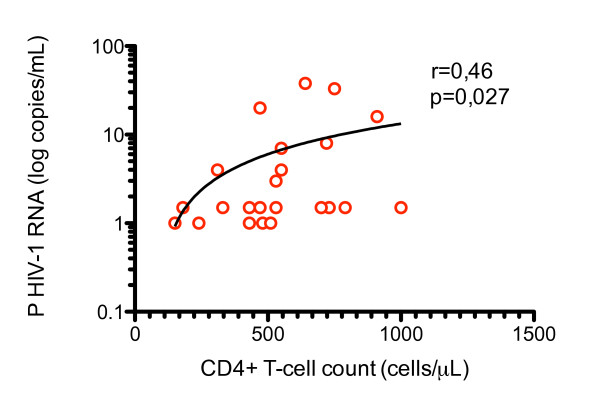
**Correlation between plasma HIV-1 RNA and CD4+ T-cell count**. Residual plasma HIV-1 RNA correlates with CD4^+ ^T-cell counts in IVIG-treated patients and controls (r = 0.46, p = 0.027).

The level of residual plasma viremia in both groups correlated with CD4^+ ^T-cell count (r = 0.46, p = 0.03), Figure 2. Nadir CD4^+ ^did not correlate to levels of plasma HIV RNA. A correlation was seen between plasma HIV-1 RNA levels and the total number of Tregs in IVIG-treated patients (r = 0.51, p = 0.02). However, when analyzing the proportion of Tregs of lymphocytes in relation to plasma viral load, no significant correlation was found. No significant correlations were found between plasma HIV-1 RNA and expression of CD38 and HLA-DR% of CD4^+ ^or CD8^+ ^T-lymphocytes. Neither were any correlations found to levels of IL-2 and IL-7 or HIV-specific CD8^+ ^T-cell responses against Gag peptide pools. No statistically significant correlation was found when comparing plasma HIV-1 RNA before antiretroviral treatment initiation to levels of residual viremia (r = 0.35, p = 0.11).

## Discussion

Residual viremia in HIV-infected patients on ART is thought to originate mainly from a small pool of latently infected resting memory CD4^+ ^T-cells[1,6,7]. As demonstrated in a recent study, viral load stabilizes within one year of treatment. However, residual low-level viremia remains measurable in a majority of patients even after as long as 7 years on suppressive therapy. The decay rate of residual viremia has been shown to be quite slow, with an estimated half-life of 9-15 months or longer[8]. Furthermore it has been established in several studies that the pool of latently infected cells is not affected by treatment intensification[9,10]. It is evident that the latent reservoir cannot be eliminated by the standard antiviral therapy currently available and this calls for alternative methods in manipulating the pool of latently infected cells. We have previously shown that the residual plasma viral load decreased in parallel with the cellular reservoir of HIV-1 in latently infected CD4^+ ^T-lymphocytes after addition of high dosage of IVIG to effective ART. Plasma HIV-1 RNA ≥ 2 copies/mL was detected in five of seven subjects at baseline, but in only one at follow-up after 8-12 weeks. These are preliminary data from a small study, with a limited number of measurements and have to be interpreted with caution. In the present study we did not measure HIV-1 in resting CD4^+ ^T-lymphocytes. However, our previous findings indicates that it is reasonable to assume that most of the plasma HIV reservoir in well-treated patients originates from CD4^+ ^resting cells. This hypothesis is also supported by the findings of Archin et al, were the level of residual viremia appears to be related to the frequency of resting CD4^+ ^cell infection[11]. Therefore a measurement of plasma HIV RNA may estimate the total plasma reservoir. The effects of IVIG treatment in general are complex and involve modulation of expression and function of Fc receptors, interference with complement activation and the cytokine network. It also generates effects on the activation and function of lymphocytes, dendritic cells, and macrophages; and provision of anti-idiotypic antibodies[12-14]. Therefore it is hard to speculate on any specific and direct mechanism involved. Moreover, it is unlikely that general immune activation results in depletion of the latent reservoir. However, we suggest that the effect of IVIG on resting CD4^+ ^T-cells is indirect and that it might be mediated by cytokines and we propose IL-7 as a possible mediator. It is known that IL-7 can activate virus expression, and it has, in conjunction with an anti-HIV immunotoxin, been shown to reduce the latent reservoir in a mouse model[15]. IL-7 also seems to induce proviral reactivation from resting T-lymphocytes isolated from HIV-1-infected patients on ART [16]. Furthermore, transient increases in plasma HIV-1 RNA levels and inducement of T-cell cycle entry has been demonstrated when administrating recombinant IL-7 to HIV-infected patients[17]. In this follow-up study, we observed a rebound of HIV-1 RNA to pre-treatment levels and no difference in plasma viral load compared to controls in median 12.4 months after IVIG-treatment. The transient effect on viral load might be explained by patients not maintaining perfect adherence allowing accidental viral replication to replenish the reservoirs. It is also possible that the IVIG effect observed in the previous study was due to a transient effect on a cellular protein or the provirus environment. Another explanation could be that ART, despite complete adherence, does not totally reduce replication, which is necessary to fully prevent new cells from getting infected. Whether the residual viremia during ART originates from ongoing rounds of replication is debated. Recent studies have revealed a significant association between low-level viremia and baseline HIV RNA levels, suggesting that the residual viremia is derived from a reservoir of long-lived cells infected before initiation of therapy[8,18]. We found no significant correlation (p = 0.11) between residual plasma viremia and levels of pre-treatment plasma HIV-1 RNA when pooling the two groups in this study. The limited number of subjects included in the present study might explain these conflicting results compared to earlier studies and this study was not powered for detection of existing possible correlation. However, we found a significant correlation when comparing plasma HIV-1 RNA with numbers of CD4^+ ^T-cells and this conflicts with earlier studies where no such correlation could be found[18,19]. If replication does occur despite ART, it is possible that a higher CD4^+ ^T-cell population may harbour additional quantities of virus. A recent study suggests an IL-7-mediated homeostatic proliferation of memory CD4^+ ^T-cells resulting in a quantitative and qualitative stability of the HIV reservoir[20]. The rebound of the residual plasma viremia that we observed in the present study may be explained by such a proliferation, an event of homeostatic forces that restores the size of the latently infected pool of resting CD4^+ ^T-cells. In our previous study a consistent increase in circulating natural Tregs was observed in all subjects after IVIG treatment. In this follow-up study we could not detect any significant difference in circulating natural Tregs in IVIG-treated patients compared to controls indicating that the increase of cells observed in the previous study was temporary during IVIG treatment. Unfortunately, we were not able to compare the Treg concentrations longitudinally over time since different methods of identification of Tregs were used in the previous and present follow-up study.

## Conclusions

We could not detect a persistent effect of IVIG treatment on the residual plasma viremia, indicating that the decrease of the latent HIV-1 pool observed in our initial study was transient, although not independently measured in this study. A correlation between HIV-1 RNA and CD4^+ ^T-cell count was found, suggesting the possibility of a larger pool of latently infected CD4^+ ^T-cells in patients with a higher CD4^+ ^T-cell count. A close correlation between the size of the latent T-cell reservoir and level of residual plasma viral load could be expected but has not been fully elucidated, although recently a small study reported that levels of residual viremia were related to the frequency of resting cell infection under suppressive treatment[11]. Further studies are needed to evaluate the immunologic effects of IVIG in HIV infection. To determine if IVIG does or does not constitute a treatment that can transiently reduce the latent HIV-1 pool, a larger, placebo controlled study would be needed.

## Competing interests

The authors declare that they have no competing interests.

## Authors' contributions

TM performed data analyses and wrote the article. VDG conducted the T-cell analysis; her supervisor was JKS. AL performed the purification and quantification of memory cells and the cytokine analyses under the supervision of AS. AE contributed to the manuscript preparation and to data analyses of the study. BS did the two-copy HIV-1 RNA PCR. MG originated the idea, designed the study, recruited the participants and performed data analysis. MG supervised the study including execution and interpretation and manuscript preparation. All of the authors contributed to the manuscript preparation and all have seen and approved the final version.
